# Renalase's Expression and Distribution in Renal Tissue and Cells

**DOI:** 10.1371/journal.pone.0046442

**Published:** 2012-10-03

**Authors:** Feng Wang, Tao Xing, Junhui Li, Mei Bai, Ruimin Hu, Zhonghua Zhao, Shoufu Tian, Zhigang Zhang, Niansong Wang

**Affiliations:** 1 Department of Nephrology and Rheumatology, Shanghai Sixth People's Hospital affiliated to Shanghai Jiaotong University, Shanghai, China; 2 The Florey Institute of Neuroscience and Mental Health, University of Melbourne, Australia; 3 Department of Pathology, Shanghai Medical College, Fudan Univesity, Shanghai, China; University of Louisville, United States of America

## Abstract

To study renalase's expression and distribution in renal tissues and cells, renalase coded DNA vaccine was constructed, and anti-renalase monoclonal antibodies were produced using DNA immunization and hybridoma technique, followed by further investigation with immunological testing and western blotting to detect the expression and distribution of renalase among the renal tissue and cells. Anti-renalase monoclonal antibodies were successfully prepared by using DNA immunization technique. Further studies with anti-renalase monoclonal antibody showed that renalase expressed in glomeruli, tubule, mesangial cells, podocytes, renal tubule epithelial cells and its cells supernatant. Renalase is wildly expressed in kidney, including glomeruli, tubule, mesangial cells, podocytes and tubule epithelial cells, and may be secreted by tubule epithelial cells primarily.

## Introduction

Renalase is a newly discovered monoamine oxidase enzyme originating from renal tissues [1]. It degrades circulating catecholamines, regulates blood pressure and cardiac function, and is closely associated with cardiovascular diseases and chronic kidney disease (CKD) [2]–[4]. Renalase is a protein made of 342 amino acids with a molecular weight of 37.8 KDa approximately. The N-terminate of renalase contains one signal peptide, one flavin adenine dinucleotide binding site and one monoamine oxidase domain, and 13.2% of its amino acid sequence is similar to the monoamine oxidase A.

Obtaining recombinant renalase protein and preparation of monoclonal antibodies are the essential steps for the study of renalase's function, expression and distribution in renal tissues and cells. Recently we have been using recombinant renalase protein produced by prokaryotic expression system to develop monoclonal antibodies, and we also have tried to use recombinant protein produced by eukaryotic expression systems such as baculovirus etc, to develop monoclonal antibodies. However, obtaining large volume of eukaryotic expressed recombinant renalase protein has not been such an easy task [5], [6].

DNA immunization technique is a vaccination method, which is a fast and effective way of stimulating body to generate immune reaction against target protein [7]–[8]. After DNA vaccine's uptake and processing by muscle cells, the natural structure of target protein remains well preserved. In recent years, DNA vaccine techniques have been used to obtain monoclonal antibodies, especially when it is difficult to get protein antigens. Recently we have successfully utilized such techniques to prepare monoclonal antibodies for retinol binding protein (RBP4) [9], and established immunological testing methods as well as explored renalase related DNA vaccine techniques [10]. On this basis, we utilized DNA immunization technique to prepare anti-renalase monoclonal antibodies and those antibodies were used to analyze renalase expression in renal tissues and cells.

## Materials and Methods

### Renalase plasmid

Primer for renalase gene was designed as Sp 5′- ATAAGAATGCGGCCGCATGGCGCAGGT GCTGATC -3′, As 5′- GGAAGATCTCTAAATATAATTCTTTAAAGCTT -3′, and then renalase gene was replicated using RT-PCR technique. Renalase gene was then inserted in pBudCE4.1 (Invitrogen, Carlsbad, CA, USA) vector plasmid, after which the immunization plasmid was completed.

### Preparation of renalase protein

Renalase protein was obtained using prokaryotic expression system. After being purified and measured for concentration, it then was stored at −20°C [5].

### Transfection of renalase plasmid

HEK293T cells purchased from ATCC (ATCC No. CRL-11268™, USA) were cultured in Dulbecco's Modified Eagle Medium (Invitrogen, USA) containing 10% fetal bovine serum. Plasmids were transfected into HEK 293T cells using Lipofectamine 2000 Reagent (Invitrogen, USA), and this process was carried out according to the manual guidelines. After 48 hours, culture media was removed and rinsed once with PBS (pH 7.4), and then dissolved (0.2 ml/hole). After the collection, the sample was heated for 5 min at 100°C, and then stored at −20°C.

### Animal immunization

Six weeks old BALB/c mice (Shanghai Experimental Animal Center of Chinese Academy of Sciences) were kept in SPF grade animal room. Animals were used according to the feeding and utilization guidelines prescribed by Chinese Academy of Science. Animal immunization was done as per the recommended methods in the literature [11]: 100 µg plasmids (100 µl sterile PBS, pH 7.4) was given intramuscularly in the quadriceps. Immediately after the injection, electric impulse stimulation was given with ECM830 (BTX, Holliston, USA). Stimulation parameters were square wave, 100 V/50 ms, positive and negative impulses given three times each, with an interval of 1 second. A total of three injections were given with an interval of 3 weeks. Three weeks after the last injection, cell fusion was carried out. A shot of intra-abdominal booster of recombinant renalase protein was given three days before the cell fusion. 10 days after the third injection, blood was withdrawn from the orbit, kept for 1 hour at the temperature of 37°C, followed by 24 hours at 4°C, and then centrifuged. Upper layer of colorless or pale yellow serum was collected and stored at −20°C for further use.

### Preparation of monoclonal antibodies

The classic method of monoclonal antibody preparation was used [12], [13]. In brief, selected one mouse that had high sensitive activation to renalase protein, harvested the spleen, then extracted spleen cells and myeloma cell line SP2/0, and then performed cell fusion with 50%(w/v)polyethylene glycol in a 50 ml centrifuge tube (Corning, New York, USA). Following the cell fusion, cells were subjected to centrifugation and transferred to 96-well plate, and then with HAT added, cell selection was performed. Ten days after the cell-fusion, cells selection was performed with selecting ELISA positive holes.

### Immunohistochemical detection of renalase's expression in renal tissues

Anti-renalase monoclonal antibodies were used as primary antibodies and SABC testing kit (Boster, Wuhan, China) was used to detect renalase's expression in renal tissues. Each testing step was performed according to the testing manual guidelines. The renal tissues subjected to testing were from a post-traumatic kidney.

### Western blot detection of renalase's expression in renal cells

With self-prepared anti-renalase antibodies used as primary antibodies and HRP labeled goat-anti-mouse IgG (Shanghai Immune Biotech, China) used as secondary antibodies, western blotting was carried out to detect renalase's expression in renal proximal tubule cells (HK2 cell line, ATCC, USA), mesangial cells (MC cell line, ATCC, USA) and podocytes (MPC5 cell line, gift from H. Xu, Children's Hospital of Fudan University, Shanghai, China).

### Immunofluoroscence detection of renalase's expression in renal tissues and cells

After the renal tissues were obtained, 3 µm thickness frozen sections or renal cell climbing slices were made and fixed with acetone, and self-prepared anti-renalase monoclonal antibodies were added as primary antibodies. Then, FITC labeled goat-anti-mouse IgG (Santa Cruz, USA) were added as secondary antibody, slide sealed with glycerol and observed under fluorescence microscope.

## Results

### Construction and validation of renalase plasmid

**Figure 1:**
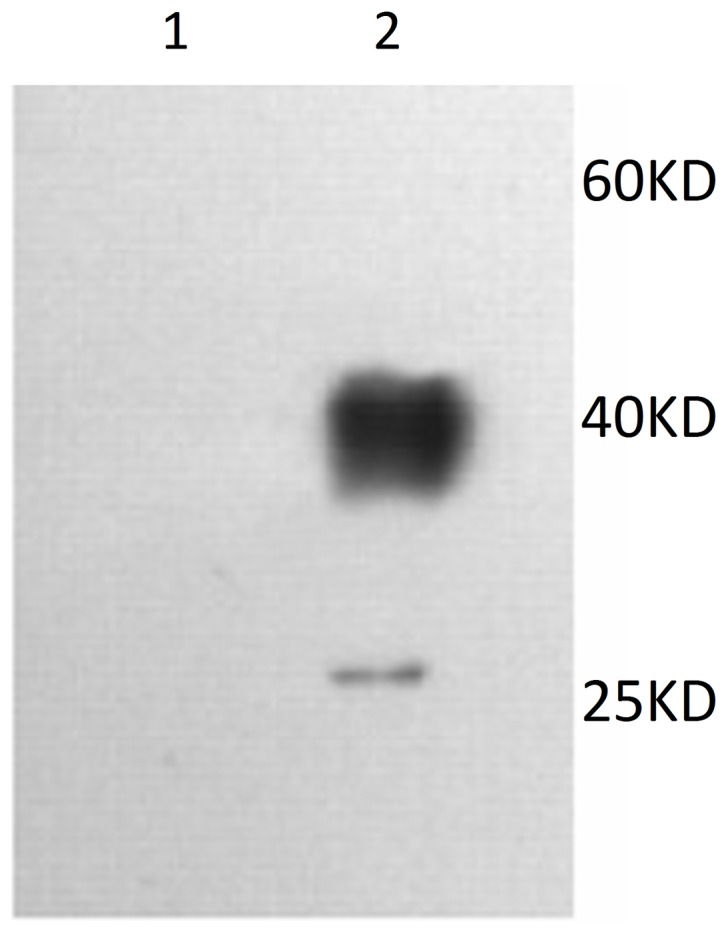
Western blotting of the expression of pBudCE4.1-Renalase in 293T cells. 1: empty pBudCE4.1vector, 2: pBudCE4.1-Renalase.

Renalase coded gene was inserted into the expression vector pBudCE4.1 (contains one His Tag gene after the sequence of multiple cloning sites), and the correct sequencing was verified. After transfection in vitro expression of pBudCE4.1-Renalase was verified by western blotting demonstrated in Fig. 1, renalase was detected by anti-His Tag monoclonal antibody. No response was observed in empty vector, indicating that renalase can be expressed in mammalian cells.

### Anti-renalase monoclonal antibody presented high sensitivity

**Figure 2:**
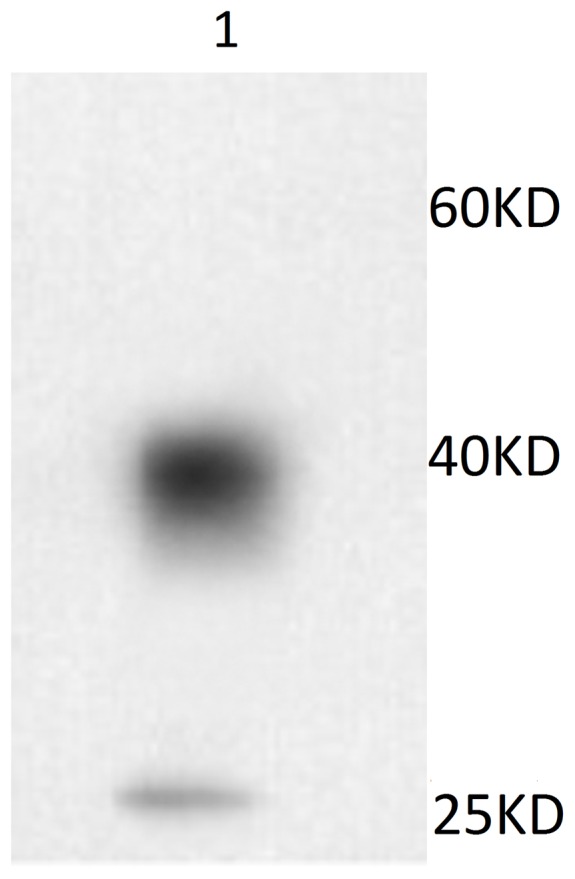
Western blotting verification of anti-renalase monoclonal antibody. 1: recombinant renalase protein.

The titer of antibody from mice serum was tested before the cell fusion. One mouse with high titer was selected as spleen cells donor and cell fusion was done with cell line SP2/0. The serum titer of anti-renalase was 1∶32000 and the titer of normal mouse was below 1∶1000. After 3 cycles of cloning, two hybridoma cell lines were obtained. Culture supernatant was collected and ascites was prepared. Analysis of culture supernatant showed an antibody titer of 1∶128000, and an antibody sub type IgG1/κ. Titration ELISA of purified anti-renalase monoclonal antibody was over 1∶512000, higher than that of poly-serum titer 1∶128000. Western blotting was performed by using prokaryotic expressed renalase protein as the sample and the prepared anti-renalase monoclonal antibodies as primary antibodies. The results showed the recombinant renalase protein can be recognized by the monoclonal antibody (Fig. 2).

### Renalase's expression in renal tissues can be detected by immunofluorescence

**Figure 3:**
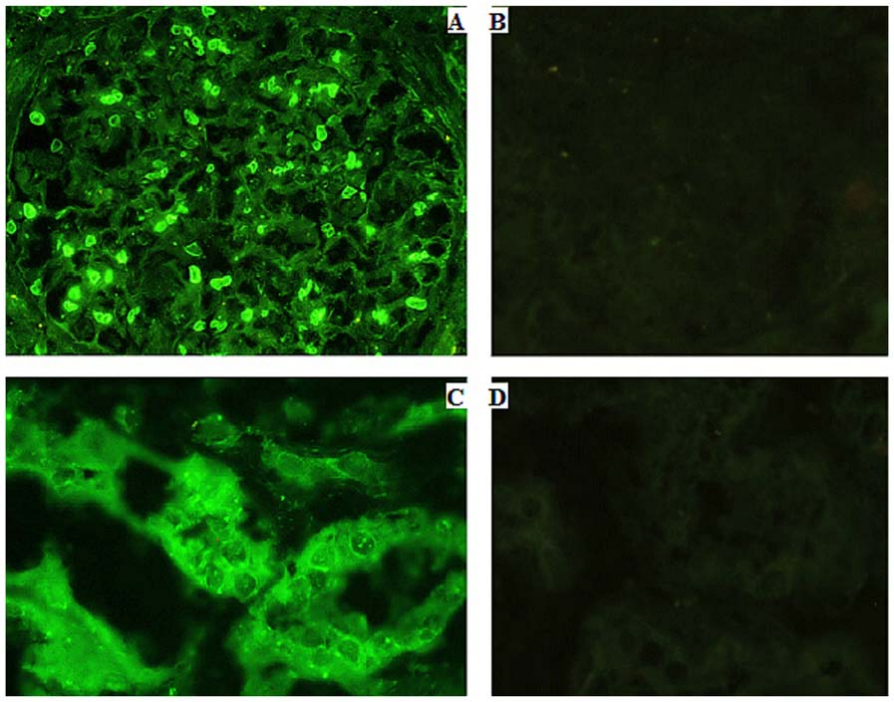
Immunofluorescence testing of renalase's expression in renal tissue. A: renalase expressed in glomeruli (Anti-renalase monoclonal antibody was used as primary antibody) (400×). B: glomeruli negative control (PBS was used as control) (400×). C: renalase expressed in renal proximal tubules (Anti-renalase monoclonal antibody was used as primary antibody) (400×). D: renal proximal tubule negative control (PBS was used as control) (400×).

Indirect immunofluorescence testing was conducted by using anti-renalase monoclonal antibody. The results showed that renalase expressed in cytoplasm of glomerular mesangial cells (Fig. 3A). And renalase can also be detected for its expression in cytoplasm of tubule epithelial cells (Fig. 3C).

### Renalase's expression in renal tissues can be detected by immunohistochemistry

**Figure 4:**
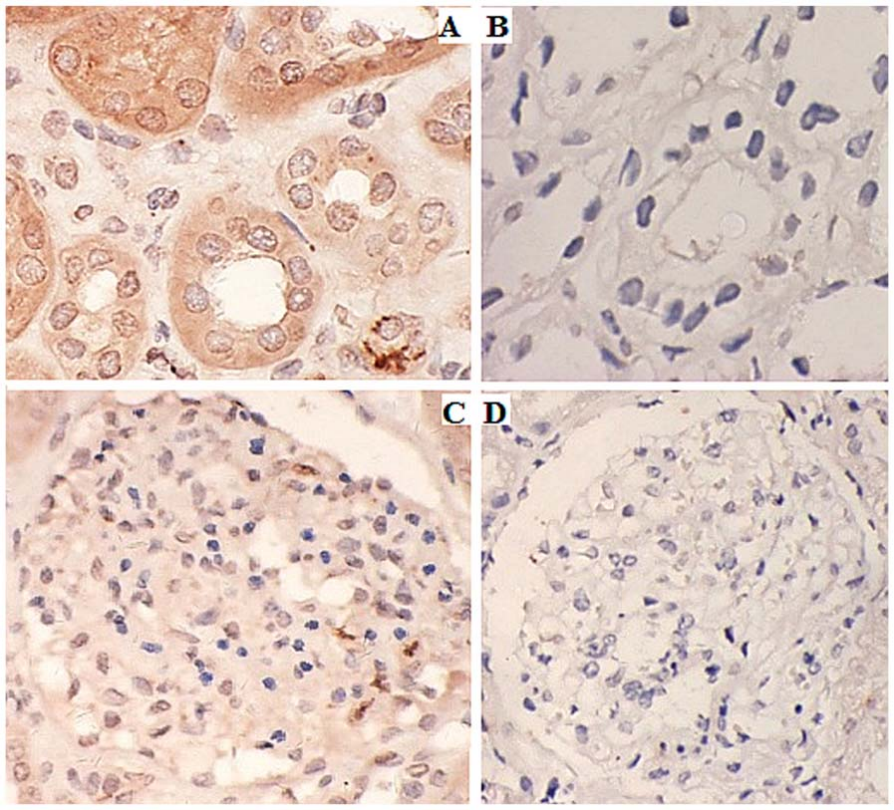
Immunohistochemical detection of renalase's expression in kidney tissues. A: renalase expressed in glomeruli (Anti-renalase monoclonal antibody was used as primary antibody) (400×). B: glomeruli negative control (PBS was used as control) (400×). C: renalase expressed in renal proximal tubules (Anti-renalase monoclonal antibody was used as primary antibody) (400×). D: Renal proximal tubule negative control (PBS was used as control) (400×).

Immunohistochemical testing was carried out with renalase monoclonal antibodies as the primary antibodies. The results showed that renalase expressed in renal glomeruli (Fig. 4A) and tubule (Fig. 4C).

### Renalase's expression in renal cells can be detected by immunofluorescence

**Figure 5:**
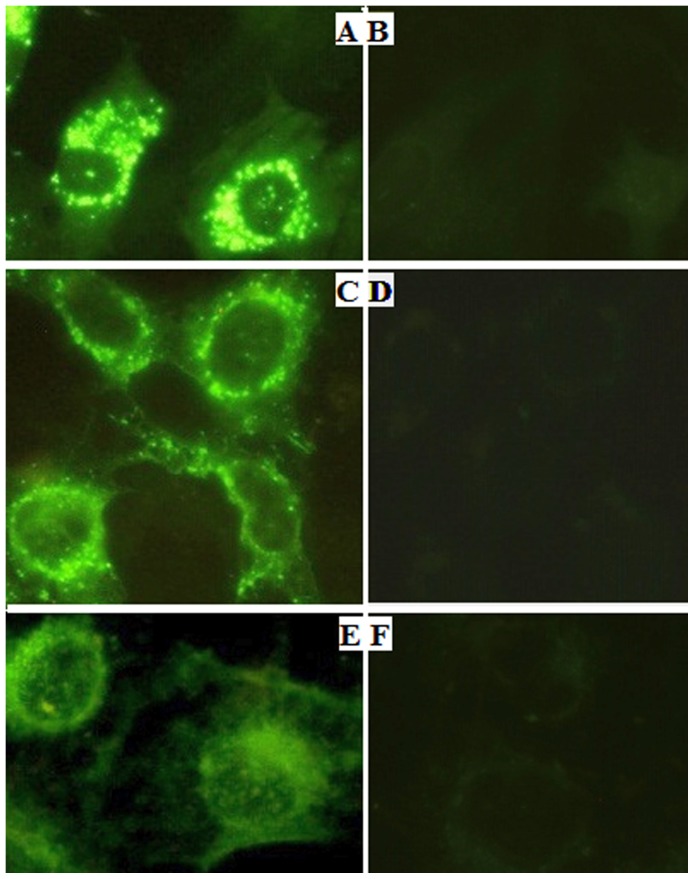
Immunofluorescence testing renalase's expression in kidney cells. A: renalase expressed in mesangial cells (Anti-renalase monoclonal antibody was used as primary antibody) (400×). B: mesangial cells negative control (PBS was used as control) (400×). C: renalase expressed in podocytes (anti-renalase monoclonal antibody was used as primary antibody) (400×). D: podocytes negative control (PBS was used as control) (400×). E: renalase expressed in renal tubular epithelial cells (anti-renalase monoclonal antibody was used as primary antibody) (400×). F: renal tubular epithelial cells negative control (PBS was used as control) (400×).

Indirect immunofluorescence testing was conducted with anti-renalase monoclonal antibodies as the primary antibodies. The results showed that renalase expressed in cytoplasm of mesangial cells (Fig. 5A), podocytes (Fig. 5C) and renal tubular epithelial cells (Fig. 5E).

### Renalase's expression in renal cells can be detected by western blotting

**Figure 6:**
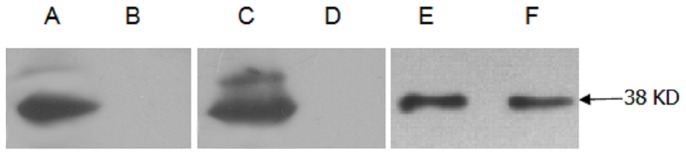
Western blotting testing renalase's expression in kidney cells. Anti-renalase monoclonal antibody was used as primary antibody. A: mesangial cells. B: culture supernatant of mesangial cells. C: podocytes. D: cuture supernatant of podocytes. E: renal tubular epithelial cells. F: culture supernatant of renal tubular epithelial cells.

Western blotting was done with anti-renalase monoclonal antibodies as the primary antibodies. The results showed that mesangial cells (Fig. 6A), podocytes (Fig. 6C) and renal tubular epithelial cells (Fig. 6E) cultured in vitro also could express renalase. But only tubular epithelial cells' supernatant could be detected the expression of renalase, as shown in Fig. 6F.

## Discussion

Recent studies showed that elevated levels of catecholamine in renal failure patients compared to normal individuals may be associated with hypertension and cardiovascular complications of CKD. But the exact mechanism of it is still unclear [14]. Renalase was discovered in 2005, it is an enzyme expressed in kidneys that can degrade circulating catecholamines. This finding has changed the previous understanding about renal physiology and neurophysiology [15]–[17]. In patients with CKD, renalase production in serum and tissues decreases. Current studies indicate that the decreased renalase production is associated with hypertension, as well as increased levels of circulating catecholamines [18]. In 2007, renalase-like substance was successfully cloned from mice by Wang [19].

Renalase is a highly conserved protein, of which 95% of amino acids are similar to that of primates. This is probably because renalase coded genes have evolved from a same ancestor gene [4]. Studies have shown that kidneys are the main source of renalase production. In vitro study has shown that renalase can degrade catecholamines and has strongest enzymatic hydrolysis action against dopamine, followed by adrenaline and noradrenaline. In vivo studies indicate that catecholamine activates renalase precursor which promotes deactivation of catecholamines and regulation of cardiovascular function [20], [21].

As of today, renalase is the only enzyme known to be secreted into blood that can degrade circulating catecholamine and may have a great value in prevention of kidney diseases and cardiovascular diseases. Discovery of renalase has drawn the attention of the scientific world [22]–[24]. Even though there have been some debates (Boomsma F), much importance is given to its significance as further studies are carried out [25]. It may have a great value in further understanding increased sympathetic activity and mechanism of cardiovascular complications in CKD patients as well as a prevention of CKD and cardiovascular diseases [26]–[28]. Both in vitro and animal studies have demonstrated the possible effects of renalase in chronic renal failure and cardiovascular diseases [29]. It can be speculated that replacement or supplementation of renalase may bring up a promising treatment.

At present, there are many ongoing studies on genetic background of renalase. Zhao et al have found that renalase gene polymorphysim is associated with primary hypertension, indicating this gene may become the novel marker of genetic susceptibility in essential hypertension [30]. Farzaneh-Far discovered that renalase gene polymorphism also is associated with ventricular hypertrophy [31]. Buraczynska found that an association between renalase gene polymorphism and hypertension in type 2 diabetes [32]. Stec et al investigated patients with end stage renal disease and found that renalase gene polymorphism is associated with hypertension among these patients [33].

Despite the role of renalase in regulating serum catecholamines and blood pressure, recent studies demonstrated that extra-renal renalase is also important. In renalase gene knocked-out mice, serum urea nitrogen, creatinine and aldosterone levels were unaffected, and cardiac systolic function was intact. However, mild ventricular hypertrophy was presented with decreased tolerance to ischemia, and increased risk of myocardial infarction, as much as 3 times higher than that of wild mice. Recombinant renalase replacement therapy could abort such abnormal changes [34]. Gu et al recently reported that renalase's expression is influenced by renal perfusion and abnormality of the renal perfusion is probably implicated in elevated serum catecholamine levels in cardiac failure [35]. Both renalase gene and protein are also observed in central and peripheral nerve systems [26]. Renalase is found in hypothalamus, pons, medulla oblongata and spinal cord, where the presympathetic and preganglionic neurons are located. It is highly plausible that renalase is also involved in regulating the central sympathetic output. Other extra-renal sources of renalase (i.e. skeletal muscles, small intestine and vasculature) are yet to be determined.

The regulation of renalase's expression is another enigma that requires further studies. As of today, there have been some animal experiments that showed decreased renalase production associated with high intake of sodium and phosphate [36]. Renal denervation, a potential anti-hypertension treatment [37], was reported to increase plasma renalase content and renalase expression in the kidneys in spontaneously hypertensive rats [38]. Data from clinical studies has shown that renalase level significantly decrease in patients with stroke and chronic kidney diseases, especially those on renal replacement therapy [39]–[41], and is normalized in kidney and heart transplant recipients [42], [43].

However what parts of kidney and which renal cell renalase is expressed in are not clear yet. In order to obtain high sensitive anti-renalase antibody, this study used DNA immunization which is an established laboratory technique that has been done successfully. Study was conducted using self-made anti-renalase monoclonal antibody, and findings were consistent with the literature, that is, renalase was expressed in both glomeruli and renal tubule [1]. Further in vitro testing showed renalase expressed in mesangial cells, podocytes and tubular epithelial cells, especially only tubular epithelial cells can secrete renalase to the supernatant. Therefore, tubular epithelial cells may be the major cells that can secrete renalase. Such observation has not been reported. These study findings indicate that anti-renalase monoclonal antobodies used in this study could identify natural renalase protein and it is a good experiment tool.

Renalase is widely expressed in glomeruli, tubules, mesangial cells, podocytes and tubular epithelial cells, but its physiological function is not clear yet. Further studies about this novel protein's molecular structure as well as function, carry a great value. At present, a lot more works need to be done on renalase's expression, regulation, function and replacement therapy etc.

## References

[1] Xu J, Li G, Wang P, Velazquez H, Yao X, et al (2005) Desir: Renalase is a novel, soluble monoamine oxidase that regulates cardiac function and blood pressure. J Clin Invest 115: 1275–1280. 15841207 10.1172/JCI24066 PMC1074681

[2] Desir GV (2008) Renalase deficiency in chronic kidney disease, and its contribution to hypertension and cardiovascular disease. Curr Opin Nephrol Hypertens 17: 181–185. 18277152 10.1097/MNH.0b013e3282f521ba

[3] Desir GV (2007) Renalase is a novel renal hormone that regulates cardiovascular function. J Am Soc Hypertens 1: 99–103. 20409839 10.1016/j.jash.2006.12.001

[4] Xu J, Desir GV (2007) Renalase, a new renal hormone: its role in health and disease. Curr Opin Nephrol Hypertens 16: 373–378. 17565281 10.1097/MNH.0b013e3281bd8877

[5] Wang F, Wang N, Xing T, Cao Y, Xiang H (2009) The cloning and expression of renalase and the preparation of its monoclonal antibody. J Shanghai Jiaotong Univ 14: 376–379.

[6] Wang F, Wang N, Xing T (2010) Construction of eukaryotic recombinant vector of renalase and its expression as a Eukaryotic Protein. J Shanghai Jiaotong Univ 15: 637–640.

[7] Tang CK, Pietersz GA (2009) Intracellular detection and immune signaling pathways of DNA vaccines. Expert Rev Vaccines 8: 1161–1170. 19722890 10.1586/erv.09.79

[8] Garren H (2009) DNA vaccines for autoimmune diseases. Expert Rev Vaccines 8: 1195–1203. 19722893 10.1586/erv.09.83

[9] Bian C, Zhang F, Wang F, Ling Z, Luo M, et al (2010) Development of retinol-binding protein 4 immunocolloidal gold fast test strip using high-sensitivity monoclonal antibodies generated by DNA immunization. Acta Biochim Biophys Sin 42: 847–853. 21062789 10.1093/abbs/gmq099

[10] Wang F, Xing T, Wang N (2011) Construction and DNA Immunization of Human renalase eukaryotic expression vector. NDT plus 4: 221. 10.1093/ndtplus/sfr021 PMC4421600 25984168

[11] Chen J, Fang F, Li X, Chang H, Chen Z (2005) Protection against influenza virus infection in BALB/c mice immunized with a single dose of neuraminidase-expressing DNAs by electroporation. Vaccine 23: 4322–4328. 15925433 10.1016/j.vaccine.2005.03.035

[12] Bian C, Zhang X, Cai X, Zhang L, Chen Z, et al (2009) Conserved amino acids W423 and N424 in receptor-binding domain of SARS-CoV are potential targets for therapeutic monoclonal antibody. Virology 383: 39–46. 18986662 10.1016/j.virol.2008.09.029 PMC7103409

[13] Zhou T, Song W, Wang F, Ni PW, Chen N, et al (2003) Cloning, expression of the lectin-EGF domain of P-selectin, and preparation of its monoclonal antibody. Sheng Wu Hua Xue Yu Sheng Wu Wu Li Xue Bao (Shanghai) 35: 172–176. 12545226

[14] Neumann J, Ligtenberg G, Klein II, Koomans HA, Blankestijn PJ (2004) Sympathetic hyperactivity in chronic kidney disease: pathogenesis, clinical relevance, and treatment. Kidney Int 65: 1568–1576. 15086894 10.1111/j.1523-1755.2004.00552.x

[15] Desir GV (2011) Role of renalase in the regulation of blood pressure and the renal dopamine system. Curr Opin Nephrol Hypertens 20: 31–36. 21099685 10.1097/MNH.0b013e3283412721

[16] Hennebry SC, Eikelis N, Socratous F, Desir G, Lambert G, et al (2010) Renalase, a novel soluble FAD-dependent protein, is synthesized in the brain and peripheral nerves. Mol Psychiatry 15: 234–236. 20168325 10.1038/mp.2009.74

[17] Desir GV (2009) Regulation of blood pressure and cardiovascular function by renalase. Kidney Int 76: 366–370. 19471322 10.1038/ki.2009.169

[18] Milani M, Ciriello F, Baroni S, Pandini V, Canevari G, et al (2011) FAD-binding site and NADP reactivity in human renalase: a new enzyme involved in blood pressure regulation. J Mol Biol 411: 463–473. 21699903 10.1016/j.jmb.2011.06.010

[19] Wang J, Qi S, Cheng W, Li W, Wang F, et al (2008) Identification, expression and tissue distribution of a renalase homologue from mouse. Mol Biol Rep 35: 613–620. 17846919 10.1007/s11033-007-9131-1

[20] Li G, Xu J, Wang P, Velazquez H, Li Y, et al (2008) Catecholamines regulate the activity, secretion, and synthesis of renalase. Circulation 117: 1277–1282. 18299506 10.1161/CIRCULATIONAHA.107.732032

[21] Ghosh SS, Krieg RJ, Sica DA, Wang R, Fakhry I, et al (2009) Cardiac hypertrophy in neonatal nephrectomized rats: the role of the sympathetic nervous system. Pediatr Nephrol 24: 367–377. 18797934 10.1007/s00467-008-0978-8

[22] Medvedev AE, Veselovsky AV, Fedchenko VI (2010) Renalase, a new secretory enzyme responsible for selective degradation of catecholamines: achievements and unsolved problems. Biochemistry (Mosc) 75: 951–958. 21073414 10.1134/s0006297910080018

[23] Paulis L, Unger T (2010) Novel therapeutic targets for hypertension. Nat Rev Cardiol 7: 431–441. 20567239 10.1038/nrcardio.2010.85

[24] Pandini V, Ciriello F, Tedeschi G, Rossoni G, Zanetti G, et al (2010) Synthesis of human renalase1 in Escherichia coli and its purification as a FAD-containing holoprotein. Protein Expr Purif 72: 244–253. 20302943 10.1016/j.pep.2010.03.008

[25] Boomsma F, Tipton KF (2007) Renalase, a catecholamine-metabolising enzyme? J Neural Transm 114: 775–776. 17385068 10.1007/s00702-007-0672-1 PMC2793395

[26] Hennebry SC, Eikelis N, Socratous F, Desir G, Lambert G, et al (2010) Renalase, a novel soluble FAD-dependent protein, is synthesized in the brain and peripheral nerves. Mol Psychiatry 15: 234–236. 20168325 10.1038/mp.2009.74

[27] Schlaich MP, Socratous F, Hennebry S, Eikelis N, Lambert EN, et al (2009) Sympathetic activation in chronic renal failure. J Am Soc Nephrol 20: 933–939. 18799718 10.1681/ASN.2008040402

[28] Malyszko J, Zbroch E, Malyszko JS, Koc-Zorawska E, Mysliwiec M (2011) Renalase, a novel regulator of blood pressure, is predicted by kidney function in renal transplant recipients. Transplant Proc 43: 3004–3007. 21996211 10.1016/j.transproceed.2011.08.032

[29] Desir G (2012) Novel insights into the physiology of renalase and its role in hypertension and heart disease. Pediatr Nephrol 27: 719–725. 21424526 10.1007/s00467-011-1828-7

[30] Zhao Q, Fan Z, He J, Chen S, Li H, et al (2007) Renalase gene is a novel susceptibility gene for essential hypertension: a two-stage association study in northern Han Chinese population. J Mol Med (Berl) 85: 877–885. 17216203 10.1007/s00109-006-0151-4

[31] Farzaneh-Far R, Desir GV, Na B, Schiller NB, Whooley MA (2010) A functional polymorphism in renalase (Glu37Asp) is associated with cardiac hypertrophy,dysfunction, and ischemia: data from the heart and soul study. PLoS One 5: e13496. 20975995 10.1371/journal.pone.0013496 PMC2958117

[32] Buraczynska M, Zukowski P, Buraczynska K, Mozul S, Ksiazek A (2011) Renalase Gene Polymorphisms in Patients With Type 2 Diabetes, Hypertension and Stroke. Neuromolecular Med 13: 321–327. 21964580 10.1007/s12017-011-8158-6 PMC3220827

[33] Stec A, Semczuk A, Furmaga J, Ksiazek A, Buraczynska M (2011) Polymorphism of the renalase gene in end-stage renal disease patients affected by hypertension. Nephrol Dial Transplant [Epub ahead of print]. 10.1093/ndt/gfr293 21617193

[34] Wu Y, Xu J, Velazquez H, Wang P, Li G, et al (2011) Renalase deficiency aggravates ischemic myocardial damage. Kidney Int 79: 853–860. 21178975 10.1038/ki.2010.488

[35] Gu R, Lu W, Xie J, Bai J, Xu B (2011) Renalase deficiency in heart failure model of rats–a potential mechanism underlying circulating norepinephrine accumulation. PLoS One 6: e14633. 21297953 10.1371/journal.pone.0014633 PMC3031511

[36] Weinman EJ, Biswas R, Steplock D, Wang P, Lau YS, et al (2011) Increased renal dopamine and acute renal adaptation to a high-phosphate diet. Am J Physiol Renal Physiol 300: F1123–1129. 21325500 10.1152/ajprenal.00744.2010 PMC3094044

[37] Esler MD, Krum H, Sobotka PA, Schlaich MP, Schmieder RE, et al (2010) Renal sympathetic denervation in patients with treatment-resistant hypertension (The Symplicity HTN-2 Trial): a randomised controlled trial. Lancet 376: 1903–1909. 21093036 10.1016/S0140-6736(10)62039-9

[38] Jiang W, Guo Y, Tan L, Tang X, Yang Q, et al (2012) Impact of renal denervation on renalase expression in adult rats with spontaneous hypertension. Exp Ther Med 4: 493–496. 23181124 10.3892/etm.2012.616 PMC3503747

[39] Malyszko J, Koc-Zorawska E, Malyszko JS, Kozminski P, Zbroch E, et al (2012) Renalase, stroke, and hypertension in hemodialyzed patients. Ren Fail 34: 727–731. 22583169 10.3109/0886022X.2012.681534

[40] Zbroch E, Malyszko J, Koc-Zorawska E, Mysliwiec M (2012) Renalase in peritoneal dialysis patients is not related to blood pressure, but to dialysis vintage. Perit Dial Int 32: 348–351. 22641741 10.3747/pdi.2011.00118 PMC3525446

[41] Zbroch E, Malyszko J, Malyszko JS, Koc-Zorawska E, Mysliwiec M (2012) Renalase, a Novel Enzyme Involved in Blood Pressure Regulation, Is Related to Kidney Function but Not to Blood Pressure in Hemodialysis Patients. Kidney Blood Press Res 35: 395–399. 22539018 10.1159/000338178

[42] Przybylowski P, Malyszko J, Kozlowska S, Koc-Zorawska E, Mysliwiec M (2011) Serum renalase depends on kidney function but not on blood pressure in heart transplant recipients. Transplant Proc 43: 3888–3891. 22172866 10.1016/j.transproceed.2011.08.075

[43] Zbroch E, Malyszko J, Koc-Zorawska E, Mysliwiec M (2012) Renalase, kidney function, and markers of endothelial dysfunction in renal transplant recipients. Pol Arch Med Wewn 122: 40–44. 22237745

